# Botany, Ethnopharmacology, Phytochemistry, and Biological Activities of *Acmella oleracea*: A Comprehensive Review

**DOI:** 10.3390/molecules31040677

**Published:** 2026-02-15

**Authors:** Ba-Wool Lee

**Affiliations:** 1 College of Pharmacy, Sahmyook University, Seoul 01795, Republic of Korea; paul36@syu.ac.kr; Tel.: +82-2-3399-1607; 2 Institute of Natural Products Science, College of Pharmacy, Sahmyook University, Seoul 01795, Republic of Korea

**Keywords:** *Acmella oleracea*, N-alkylamides, spilanthol, essential oil, phenolic compounds, quantitative analysis

## Abstract

*Acmella oleracea* (L.) R. K. Jansen (Asteraceae), commonly known as the “toothache plant” or “jambu,” is a significant medicinal plant that has been traditionally used in Brazil and other tropical and subtropical regions for relieving dental pain, as an anti-inflammatory agent, and as a culinary spice. Due to its versatile utility, this plant has been extensively studied in modern medicine and pharmacy for its diverse pharmacological properties, including anesthetic, analgesic, anti-inflammatory, antioxidant, and antimicrobial activities. Analytical research on the chemical compositions responsible for these activities has led to the identification of approximately 120 secondary metabolites. These findings provide scientific validation for its traditional uses and have spurred research into the development of ingredients for functional foods and cosmetics. This review incorporates the latest research findings, focusing on biological activities and compounds that have been practically isolated or can be isolated based on quantitative experimental data, to serve as a practical reference for industrial development. Furthermore, factors influencing the content of alkylamides and phenolic compounds, two major bioactive groups, are summarized to support material development. Ultimately, this review aims to provide a clearer understanding of the plant’s utility and contribute to the development of products that enhance human health.

## 1. Introduction

**Figure 1:**
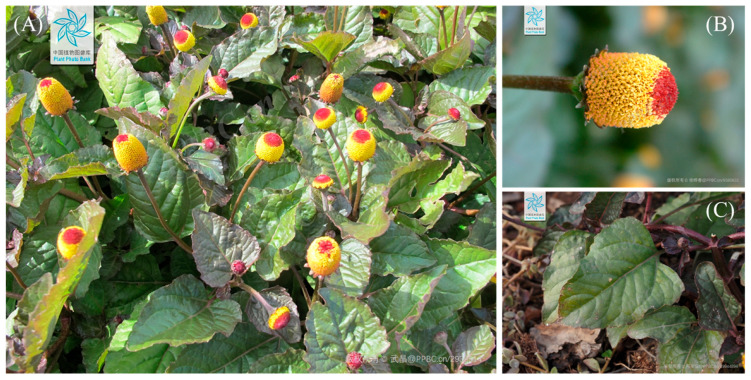
Morphological image of *A. oleracea*. (**A**) Aerial part, (**B**) flower with pedicel, (**C**) leaf with petiole. (The images were obtained from the Plant Photo Bank of China (PPBC), an open scientific resource. The watermarks on the images indicate the copyright holders and the source identification numbers).

*Acmella oleracea* (L.) R. K. Jansen (*A. oleracea*), a member of the Asteraceae family, is an annual herb characterized by its yellow-to-red cylindrical discoid capitula. Due to the distinct appearance of its central discoid, which turns red, it is commonly referred to by various names such as eyeball plant, spot flower, and buzz button (Figure 1). More notably, it is widely known as the “toothache plant” owing to its exceptional ethnomedicinal efficacy in relieving dental pain. It is also called “paracress,” a term derived from the Para region of Brazil, its place of origin, meaning “cress-like vegetable.” [1,2]. Beyond its use for toothaches, *A. oleracea* has been traditionally utilized for a diverse range of medicinal purposes. Various parts of the plant have been employed to treat wounds, stomachaches, skin diseases, and muscular pain, or serve as laxatives, anthelmintics, and appetite enhancers [3]. Reflecting these broad ethnopharmacological applications, extensive modern scientific research has been conducted on its pharmacological activities, including anti-inflammatory, antioxidant, analgesic, anesthetic, antiplasmodial, antibacterial, wound healing, and antipyretic effects. In addition to academic interest, there is active research into its development as functional health foods. In particular, spilanthol, the representative bioactive compound of this species, has seen a significant increase in patent registrations for technological development. Its applications are being widely explored in oral care products, personal care items, detergents, and the food and beverage industry [4]. Consequently, *A. oleracea* remains a botanical resource with substantial potential for further exploration. To facilitate effective research and industrial application, a systematic consolidation of existing research findings is highly warranted. Although there have been relevant review papers, they mainly focus on either specific chemical classes, the entire *Acmella* genus, or a particular viewpoint [2,5,6]. This review aims to provide the most up-to-date and comprehensive analysis of the phytochemical and pharmacological profiles specifically for *A. oleracea*. In terms of phytochemical reporting, not all compounds detected by mass spectrometry or other spectroscopic methods were included; rather, only comprehensively summarized compounds that have been directly isolated or reported with quantitative data in articles were included, ensuring the information is robust enough for actual industrial development. This review involved a thorough assessment of reported doses (and administration routes), experimental models, and outcomes. Additionally, it scrutinized the plausible mechanisms of action associated with *A. oleracea* and its primary bioactive compounds.

## 2. Methodology

The valid scientific names and synonyms obtained from the International Plant Names Index (IPNI), with reference to the WFO Plant List, Kew, and Flora of China, were used for the literature search. A literature search was conducted in PubMed/MedLine, Science Direct, and Google Scholar. The search and data collection were performed using the primary keyword *Acmella oleracea*, *Spilanthes acmella*, a term frequently misused by many researchers, as well as phytochemical, activities/effects, bioavailability, clinical, and isolation. No restrictions were placed on the publication year of literature. However, to accommodate space limitations and ensure effective description, we prioritized the most recent or most significant studies when multiple papers reported on the same activity with only minor variations, except in special cases. The search was limited to English. An image of geographical distribution of the plant was downloaded from the Global Biodiversity Information Facility (GBIF) and further checked with the available literature.

## 3. Botany

The genus *Acmella*, a member of Asteraceae (conserved name, Compositae), embraces 30 species and nine additional infraspecific taxa dispersed in tropical and subtropical areas [7] (Table 1). Since the genus shows extremely complicated patterns from a morphological and chromosomal perspective, it has been very difficult to demarcate the taxa and has often been misapplied in either description or illustration [8]. Although there has been reclassification and restoration of the taxonomical position of the genus *Acmella* by means of cladistic and chromosomal studies [7], it is sometimes mentioned interchangeably with the genus *Spilanthes*, even in recently published papers. *A. oleracea*, the most distinguished and recognized species for its medicinal purpose, is sometimes described as identical to *Spilanthes acmella* (L.) Murr. which has the officially accepted name *Blainvillea acmella* (L.) Philipson ([7], World Flora Online, 2025).

**Figure 2:**
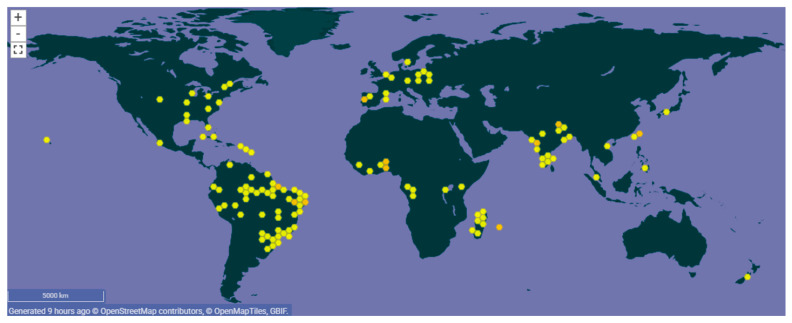
General geographical distribution of *A. oleracea* in the world (www.gbif.org) (accessed on 5 February 2026).

Although its native habitat is unclear, many reported that it originated from Amazonian countries in South America such as Peru and Brazil as the majority opinion [1,9]. Jansen suggested that *A. oleracea* was highly likely cultivated from *A. alba*, a wild plant in Peru [7]. From these countries, it has been introduced to and subsequently cultivated in many other countries within tropical and subtropical regions, such as the United States, Mexico, India, Taiwan, Germany, France, and Madagascar, which is supported by its various vernacular names from many different countries [1,10,11] (Figure 2).

As an annual herb, the height of *A. oleracea* reaches up to 20–90 cm high, and its stems are commonly decumbent or erect, but burly, branched and glabrous with green to red. Petioles are 2–6.5 in length and glabrous or barely pilose, and it has winged stipules in its base [1,7]. For leaves, the shape of leaf blade is generally broadly ovate with an acute apex, and that of the margin is dentate, with 5–10 cm and 4–8 cm for length and width, respectively. Both surfaces of leaf are hairless, and its base truncates or shortly attenuates. Inflorescence is solitary but sometimes axillary. The capitula (a dense cluster of many small flowers), the most distinct feature of *A. oleracea*, is cylindrical discoid-shaped with 10.5–23.5 mm and 11–17 mm measurements for height and diameter, respectively [1,7]. Moreover, the color is featured golden-yellow with a prominent dark red center. Since it resembles an eye, one of its nicknames is eyeball plant. The peduncles, a small stalk that joins the flower to the main stem, is glabrous or barely hairy with a 3.5–12.5 mm length [1,7] (Figure 1).

## 4. Traditional Uses

*A. oleracea*, believed to have originated in Peru or Brazil, has been introduced and utilized in numerous countries worldwide due to its ease of cultivation and diverse therapeutic properties. Its primary applications are medicinal and culinary, while it also serves minor roles in tribal religious rituals and as an ornamental plant [12,13].

### 4.1. Medicinal Purpose

The most prominent global medicinal application of *A. oleracea* is for the relief of toothache and as a local anesthetic. For instance, among the Irula tribe in the Hasanur Hills of Tamil Nadu, India, where the plant is locally known as “Mandal Poo Chedi,” the flowers are crushed and applied directly to the affected site [14]. This extensive use for treating toothache, throat infections and gum infections across the Americas, Asia, and Africa has earned the species its well-known nickname, the “toothache plant” [6,14,15].

In Brazil, the plant is commonly referred to as “jambu” and has been traditionally used by inhabitants of the Amazon basin to treat tuberculosis. Also, it is marketed as a female aphrodisiac at the Vero-Peso fair, a local market in the city of Belém, the largest city in the Amazon region, Pará [16]. In India, *A. oleracea* has been renowned as the most prominent agent to improve the sexual functions of men in the traditional Ayurvedic system [17]. Specifically, in the Chhindwara district of Madhya Pradesh, a paste made from the roots is employed to treat various throat problems [18]. Furthermore, the flowers are recognized for their sialogogic effect (inducing salivation), which is utilized to alleviate xerostomia and aid digestion—an effect primarily attributed to the presence of spilanthol [19]. In some cultures, the flowers are also used to treat stammering in children [20].

Regional variations in the medicinal application of *A. oleracea* are diverse and culturally specific. In the Bogra district of Bangladesh, the plant is locally referred to as “Vhadalika”, where its leaves and flowers are traditionally employed to treat leucorrhea in women [21]. Similarly, in the Chittagong Hill Tracts of Bangladesh, it is known as “Jhummosak”, and a paste derived from the entire plant is utilized as a remedy for poisonous stings [22]. In Cameroon, the species serves a dual purpose as a treatment for snakebites and a remedy for articular rheumatism [23]. Besides these, the roots of the plant have been utilized as a purgative to relieve constipation, and the whole herb has been used to facilitate the expulsion of urinary calculi (urolithiasis) and to treat conditions such as scurvy and various digestive disorders [24].

### 4.2. Culinary Uses

Thanks not only to its medicinal value, but also to the unique tingling and pungent sensations they produce, the leaves and flowers of *A. oleracea* are integral to various culinary traditions. The culinary applications of *A. oleracea* are geographically diverse and reflect the plant’s unique sensory profile. In the United States, the raw leaves are utilized as a pungent flavoring agent in salads [25], whereas in India, they are typically prepared as a cooked vegetable for inclusion in soups and meat dishes [7,26]. In Brazil, both fresh and cooked leaves serve as staple ingredients in Indigenous cuisine, particularly within the provinces of Acre, Amazonas, Pará, and Ceará [27]. Culinary traditions in Southeast Asia also incorporate the plant; in Java, Indonesia, young shoots and leaves are served raw as ‘lalab’ accompanied by sambal, while in Thailand, both the leaves and flower heads are integrated into various curries [28,29]. Furthermore, in Japan, the flowers are valued as a food spice and are also employed as a flavoring agent in dentifrices [30].

The flower buds, commercially known as “Buzz Buttons”, “Szechuan Buttons”, or “Electric Buttons”, are often used to offset the intense heat of chilies [27,31]. Ingesting a whole bud results in an initial grassy flavor, followed by an intense numbing sensation, excessive salivation, and a cooling effect in the throat. Notable modern culinary applications include Szechuan Button-infused eel dishes, cocktails, and sorbets [27,31]. In India, the buds and their oleoresins are also used to flavor chewing tobacco. Related information is summarized in Table 2.

## 5. Phytochemistry

Due to the obvious and various activities of *A. oleracea*, phytochemical composition of the plant has been extensively studied. Some GC/MS-based chemical studies have reported that *A. oleracea* is rich in essential oil, comprising about 15% of the constituents among which alkylamides (**1**–**22**), monoterpenoids (**23**–**28**), sesquiterpenoids (**29**–**37**) are present dominantly [35,36]. Also, some other terpenoids such as a diterpene (**38**) and triterpenes (**39**–**43**), and some lipophilic constituents such as steroids (**60**–**63**) and hydrocarbon derivative (**64**–**72**), have also been reported from the plant [20,37,38]. Not only these lipophilic parts, but also hydrophilic fractions, began to attract attention because of activity-guided isolation. As opposed to inflorescences, leaves and root are rich in phenolic compounds (**44**–**56**, **59**) and flavonoids (**57**, **58**) [39,40]. Major second metabolites which have been isolated or identified with high abundance from various studies are summarized in Table 3.

### 5.1. N-Alkylamides

**Figure 3:**
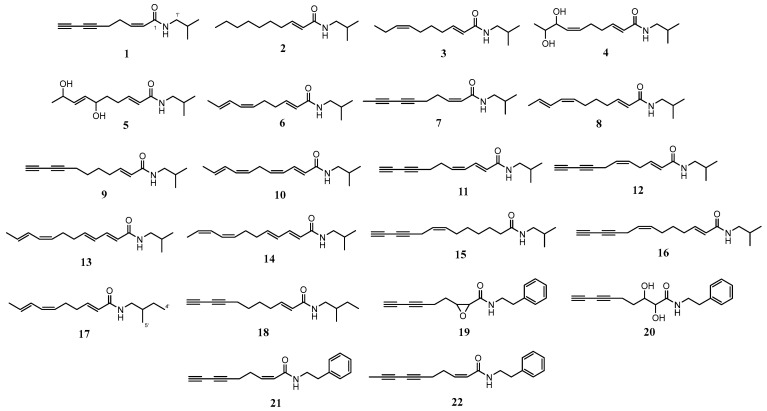
Structures of N-alkylamides in *A. oleracea*.

N-Alkylamides (NAAs) are characterized by a molecular structure centered around an amide bond, flanked by a fatty acid chain on one side and a residual amino acid moiety on the other. As expected from the structure, it is biologically synthesized by a condensation reaction of a fatty acid chain (unsaturated in most cases) and amin moiety derived from amino acids through decarboxylation [42]. Through the numerous combinations of 200 fatty acids and 23 amines, more than 300 NAAs have been reported from a total of 25 plant families, mainly eight (Asteraceae, Piperaceae, Rutaceae, Brassicaceae, Euphorbiaceae, Aristolochiaceae, Menispermaceae, and Poaceae) [58,59]. Though some families such as Convolvulaceae, Euphorbiaceae, Menispermaceae, and Rutaceae show the biosynthesis of NAAs featuring aliphatic moieties in both their amine and acid components as a prominent metabolic characteristic, they also produce other major types of secondary metabolites including alkaloid [59]. On the other hand, several genera in Asteraceae, such as *Acmella*, *Spilanthes*, *Echinacea* and *Heliopsis*, dominantly produce NAAs as major metabolites, conferring their distinct properties [60]. As NAAs containing plants generally exhibit a pungent taste, numbness, and tingling sensation [40], NAAs, especially spilanthol (**6**) (also called affinin), which is one of the most famous NAAs in Asteraceae, became the reason for many vernacular names of *A. oleracea* such as ‘toothache plant’ and ‘brede mafane,’ which means hot taste grass in Malagacy [40,61]. NAAs from the *Acmella* genus are normally composed of N-isobutyl, N-methylbutyl or N-phenethylamine for the amine part and medium-chain residues (C_8_ to C_13_) for fatty acid [60]. Among 70 NAAs identified from *Acmella* and *Spilanthes* species [42], 22 NAAs (**1**–**22**) have been isolated or identified with moderate or large amounts sufficient to be isolated from *A. oleracea*, and many of them are N-isobutylamides (**1**–**16**), followed by N-phenethylamides (**19**–**22**) and N-methylbutylamides (**17** and **18**) in the order of frequency (Figure 3 and Table 3). The stereochemistry of **4**, **5** and **20** was not determined in the corresponding references.

Since many biological activities were known to be ascribed to NAAs, there have been various research efforts towards the development of efficient extraction methods or quantification for NAAs, especially spilanthol (**6**) for industrial purposes. As NAAs are normally amphiphilic due to the relatively polar amide and lipophilic acyl chain residue easily expected from the structure, a wide range of solvents such as methanol, ethanol, n-hexane, ethyl acetate, or even CO_2_ for superciritical extraction have been used [17,36,46,62,63]. Ferrara reported that NAAs are accumulated five times more in aerial parts (2.77 mg/g, DE) than root (0.49 mg/g, DE), and for spilanthol (**6**), they are ten times more concentrated in aerial parts (2.20 mg/g, DE) than root (0.22 mg/g, DE), which is also supported by reports on isolation of NAAs (Table 3). When limited to aerial parts, NAAs in flower and leaf parts are similar at 7.69% and 8.42%, respectively, which are twice as much as that in the stem (3.09%) [64]. Meanwhile, the content of spilanthol (**6**) was much higher in the flower part (16.50 mg/g, DW) than in leaf (0.34 mg/g) and stem parts (0.24 mg/g) in the accelerated solvent extraction method. In a separate study, Dias et al. showed that the yield of spilanthol (**6**) in flower, leaf and stem were 65.4, 19.7 and 47.3%, respectively, by means of supercritical fluid extracted coupled enhanced solvent extraction (SFE-ESE) method, demonstrating that low content of spilanthol (**6**) in the leaf and stem part could be improved to some extent depending on the extraction method [46]. In the case of optimization of the extraction solvent, Kavallieratos et al. evaluated extraction efficiency of various solvents (n-hexane, ethanol, methanol, dichloromethane, petroleum ether, ethyl acetate) for spilanthol (**6**) contents by using sonication and concluded that n-hexane extract showed the most concentrated content of spilanthol (**6**) (20.9 g/100 g, dry extract), followed by petroleum ether (19.7), dichloromethane (17.7), ethyl acetate (16.5), methanol (15.9), and ethanol (11.4), but methanol extract exhibited the largest absolute amount (1.3 g/100 g, dry biomass), followed by dichloromethane (0.9), ethyl acetate (0.7), n-hexane (0.6), ethanol (0.4), and petroleum ether (0.4) [62]. Meanwhile, another study reported that absolute amounts of spilanthol (**6)** in a defatted 80% ethanol fraction and n-hexane fraction were similar after consideration of concentration and extraction yield in in vitro seedlings [40].

For the quantitative analysis of major NAAs, spilanthol (**6**) has the highest proportion in flower, while it was contained in medium or small amounts when limited to the leaf part, though it is the most abundant NAA in the whole aerial part [36,48,62]. Besides spilanthol (**6**), (2E,6Z,8E)-N-(2-methylbutyl)-2,6,8-decatrienamide (homospilanthol) (**17**), (2E,7Z)-N-isobutyl-2,7-decadienamide (**3**), (2E)-N-isobutyl-2-undecene-8,10-diynamide (**9**), (2E,7Z)-N-isobutyl-2,7-tridecadiene-10,12-diynamide (**16**), and (2E)-N-(2-methylbutyl)-2-undecene-8,10-diynamide (**18**) have been reported in medium or small amounts enough to be isolated in whole aerial part [36,48,61,62]. Interestingly, there was an attempt to develop an optimized extraction method maximizing the content of spilanthol (**6**) in essential oil, and reporting microwave-assisted extraction showed around six times higher yield (13.31 g/100 g in EO) than that of hydrodistillation (2.24). In most cases, fatty acid moiety in NAAs is unsaturated, so it is vulnerable to sunlight and easily degraded. Bearing in mind the deleterious effect on usefulness of *A. oleracea*, Savic et al. conducted well-timed research on the stability of the main NAA in commercially purchasable products. Photostability of **1**, **6**, **9**, **11**, **16**, **17**, **18**, **19** and **21** in hydroglycolic extract of *A. oleracea* suspended in various solvents (methanol, ethanol, saline, water) was evaluated in 30 min intervals up to 120 min. As a result, (2E,6Z,8E)-N-(2-methylbutyl)-2,6,8-decatrienamide (homospilanthol) (**17**) and spilanthol (**6**) turned out to be the most stable NAAs in order. Also, in methanol, there were no significant differences in stability among compounds throughout time, whereas in other solvents, stability decreased significantly in the order of ethanol, saline, and water. Compounds **4** and **5** were suggested as degraded forms of spilanthol (**6**) [51]. The research findings regarding the contents of spilanthol (**6**) and NAAs according to various factors such as plant parts, extraction methods, and solvents are summarized in Table 4.

### 5.2. Terpenoids

**Figure 4:**
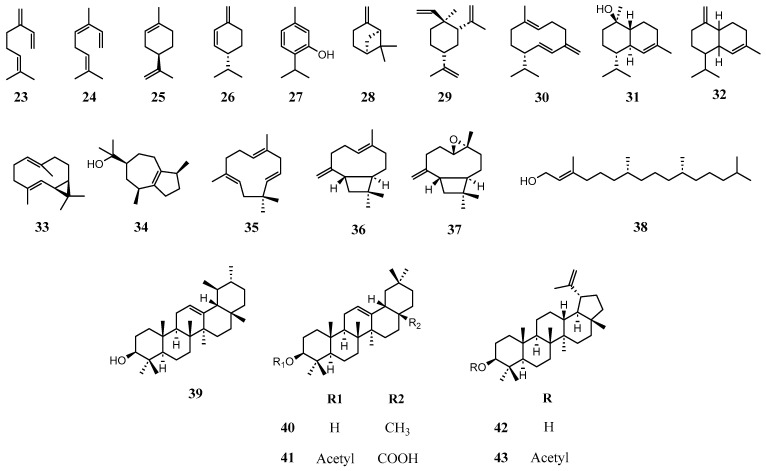
Structures of terpenoids in *A. oleracea*.

Terpenoids, the largest and most structurally diverse group of plant secondary compounds, are biosynthetically constituted by assembly of 5-carbon isoprene units (C_5_H_8_) and further categorized as several subgroups such as monoterpene, sesquiterpene, diterpene, and triterpene according to the number of attached isoprene units [65,66]. In total, 21 terpenoids were isolated or identified with isolatable amounts from *A. oleracea*, including six monoterpenoids (**23**–**28**), nine sesquiterpenoids (**29**–**37**), one diterpenoid (**38**), and five triterpenoids (**39**–**43**) (Figure 4 and Table 3). Regarding the absolute configuration of compounds **30**, **33**, and **34**, although their stereochemistry was not explicitly confirmed in the cited literature, the forms that predominantly exist in nature, particularly in essential oils, were adopted [67,68,69]. In contrast, the stereochemistry of **32** was not reported in the original studies, and this compound is known to exist in various isomeric forms in nature. Apart from the representative compound, spilanthol (**6**), most phytochemical studies on *A. oleracea* have focused on the monoterpenes and sesquiterpenes that constitute its essential oil. Essential oil of *A. oleracea* is recently attracting attention due to its varying biological activity, especially insecticidal activity. Many researchers have reported the results of quantitative and qualitative analysis of components of essential oil in different parts of the plant and by different extraction methods. Most of the analyses on essential oil and chemical identification were performed by comparison with analytical standards, mass spectrum overlapping or retention index calculation, and comparison with those in well-established libraries such as ADAMS, FFNSC3, NIST17 by means of GC-MS [35,36,45]. Monoterpenes and sesquiterpenes are two major components in the essential oil of *A. oleracea* constituting around 90% of the oil, with minor hydrocarbon and fatty alcohol. In accordance with commonly observed patterns, monoterpenes are more abundant than sesquiterpenes [9,35,70]. However, the monoterpene is majorly composed of monoterpene hydrocarbon rather than oxygenated monoterpene which is opposite to general cases [71]. Many studies reported major compounds in the essential oil of *A. oleracea*.

Among these, Baruah and Leclercq investigated chemical composition of essential oil of flower head of *A. oleracea* in India by steam distillation and reported limonene (**25**) (23.6%), (Z)-β-ocimene (**24**) (14.0%) and myrcene (**23**) (9.5%) for monoterpene, and β-caryophyllene (**36**) (20.9%) and germacrene D (**30**) (10.8%) for sesquiterpene, as major constituents out of 30 detected compounds [52]. In another study utilizing same extraction method for the leaves and stem of *A. oleracea* in Brazil, thymol (**27**) (18.3%) and γ-cadinene, (**31**) (13.3%) along with β-caryophyllene (**36**) (30.2%), were reported as major constituents [53]. In separate studies utilizing the hydrodistillation method, pinene (**28**) (17.3%) and caryophyllene oxide (**37**) (10.0%) were identified as additional major compounds extracted by hydrodistillation from the flower of *A. oleracea* harvested in Italy, and β-elemene (**29**) and bicyclogermacrene (**33**) were also identified with isolatable amounts from leaves of *A. oleracea* [9,54]. Interestingly, Jerônimo et al. reported qualitative and quantitative analysis of components in the essential oil of *A. oleracea* in a comparison of two conventional extraction methods (hydrodistillation versus steam distillation) and two different parts (flower versus leaf) [35]. Total yield of essential oil from flower by hydrodistillation (0.68%) was superior to that by steam distillation (0.5%). Moreover, for most of the major constituents, yield by hydrodistillation was much higher than that from steam distillation. However, 38 out of 62 were detected by hydrodistillation, while 49 were detected by steam distillation. In many other plants, total yield of essential oil is higher when it is extracted by hydrodistillation, and the number of identified compounds by steam distillation is higher, though there are some plants which showed much higher total yield by steam distillation, and some compounds that could be extracted solely by that method [72,73]. β-pinene (**28**), myrcene (**23**), β-phelandrene (**26**), and guaiol (**34**) were solely found in the flower part. Including minor compounds, most of the compounds were found either in both the flower and leaf or only in the flower part. In addition to the two conventional methods, microwave-assisted extraction method is becoming popular due to its effectiveness in time and yield. Spinozzi et al. reported higher total yield of essential oil by microwave-assisted extraction as 0.47% along with maximized spilanthol (**6**) content (13.31%) when compared to that by hydrodistillation (0.22% for essential oil and 2.24% for spilanthol (**6**)) [36].

As seen in the previous reports, there are various factors affecting the yield and chemical composition of essential oil such as part of plant extracted, method of extraction, and cultivation environment [9,74,75]. Regardless of all these influential factors, certain terpenes, specifically E-caryophyllene (**36**), germacrene D (**30**) for the aerial part including the flower and myrcene (**23**), and β-pinene (**28**) majorly in the flower, consistently serve as distinctive chemical markers for *A. oleracea* oils, as evidenced by the existing literature. In addition to these constituents comprising essential oil, (E)-phytol (**38**), a diterpene, was identified in isolatable amounts through GC-MS, and some triterpenes such as α-amyrin (**39**), β-amyrin (**40**), 3-acetylaleuritolic acid (**41**), lupeol (**42**), and 3-acetyllupeol (**43**) were isolated [11,37,39,55].

### 5.3. Phenolic Compounds

**Figure 5:**
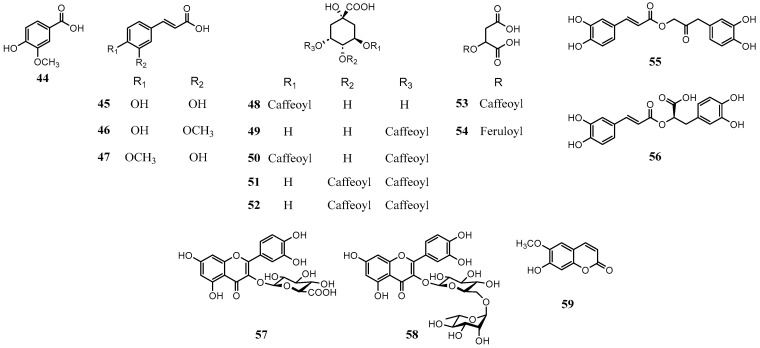
Structures of phenolic compounds in *A. oleracea*.

Phenolic compounds are defined by the presence of one or more hydroxyl groups directly attached to an aromatic ring, with the entire classification centered around the structure of phenol [76]. Thus, the term ‘phenolics’ encompasses a broad spectrum of chemical substances such as cinnamic acid derivatives, flavonoids, coumarins and lignans, which were categorized based on the number of carbons by Harborne and Simmonds [77]. Phenolic compounds have long been recognized as vital therapeutic agents for non-communicable diseases and lifestyle disorders, including cardiovascular diseases, various cancers, and age-related pathologies due to their potent antioxidant capacity to stabilize free radicals through hydrogen and electron donation [78]. Consequently, numerous studies have been reported to quantify the phenolic content and evaluate the antioxidant potential of *A. oleracea*. Perhaps because they have been overshadowed by alkylamides, there are surprisingly few reports on the actual isolation of individual phenolic compounds from *A. oleracea*. Instead, the literature has predominantly focused on the determination of total phenolic and flavonoid contents, as well as the preliminary identification of phenolic constituents. Based on the evidence from actual isolation and data confirming quantitative correlations during characterization, the structures of the identified phenolic compounds are illustrated in Figure 5. The reported compounds include vanillic acid (**44**), a simple phenolic acid (C_6_-C_1_); cinnamic acid derivatives (**45**–**56**) (C_6_-C_3_); flavonoid glycosides (**57** and **58**) (C_6_-C_3_-C_6_); and coumarin (**59**) (Table 3).

The extraction efficiency of total phenolic contents (TPC) from *A. oleracea* is influenced by various physicochemical factors. Satao et al. conducted a comprehensive kinetic study focusing on solvent type, solid-to-solvent ratio, temperature, agitation speed, and pH. Regarding solvent selection, although methanol provided the highest yield (20.20 mg GAE/g DM), water was identified as the optimal solvent (17.98 mg GAE/g DM) [79]. This choice was justified by water’s safety for food and pharmaceutical applications, its economic viability, and its low vapor pressure, which facilitates the maintenance of a stable solid-to-solvent ratio by minimizing evaporation.

Optimization of other parameters revealed that a 1:30 solid-to-solvent ratio was ideal, as yields plateaued beyond this point (18.52 to 18.60 mg GAE/g DM). Temperature studies showed a sharp increase in TPC up to 50 °C (18.21 mg GAE/g DM), followed by a decline at 60 °C, likely due to the thermal degradation of sensitive polyphenols. Furthermore, extraction was most effective at pH 5.0, with yields decreasing significantly as conditions became alkaline (pH 6–8). This aligns with the findings of Tsao et al., who noted that acidic conditions maintain polyphenols in a neutral state, thereby enhancing their solubility [80]. While Satao et al. recommended an agitation speed of 400 rpm, the data suggests that a plateau was not definitively reached, and higher speeds, such as 500 or 600 rpm, might yield even more favorable results.

From a biological perspective, Nascimento et al. investigated the distribution of phenolics and flavonoids across different plant parts and cultivation systems [56]. Regardless of whether the plants were field-grown (FG) or hydroponically grown (HG), leaves exhibited the highest phenolic content, followed by flowers and stems—a trend consistent with reports by Abeysiri et al. [10]. In terms of growth systems, FG plants generally outperformed HG plants in TPC. This contrasts with studies by Abeysinghe et al., where HG plants and callus cultures (CC) showed higher yields [63]. Such discrepancies may stem from variations in analytical methodologies, hydroponic techniques, or geographic cultivation regions.

Further characterization by Ferrara compared the aerial parts (AP) and roots (R) [43]. While AP extracts were rich in both phenols and alkylamides (including spilanthol (**6**)), R extracts contained significantly higher phenolic levels (14.15 mg/g DE) despite much lower alkylamide content. Notably, the neuroprotective effect of the root extract was comparable to that of the aerial parts, suggesting that phenolic compounds play a crucial role in this bioactivity. The root profile was dominated by 3,5-di-O-caffeoylquinic acid (**51**), accounting for over 50% of its TPC, whereas the aerial parts contained a more diverse but lower-concentration mixture of phenolics, including caffeoylmalic acid (**53**) and feruloylmalic acid (**54**). Detailed data on these phenolic constituents and the factors influencing their extraction are summarized in Table 5.

### 5.4. Steroids and Other Lipophilic Compounds

**Figure 6:**
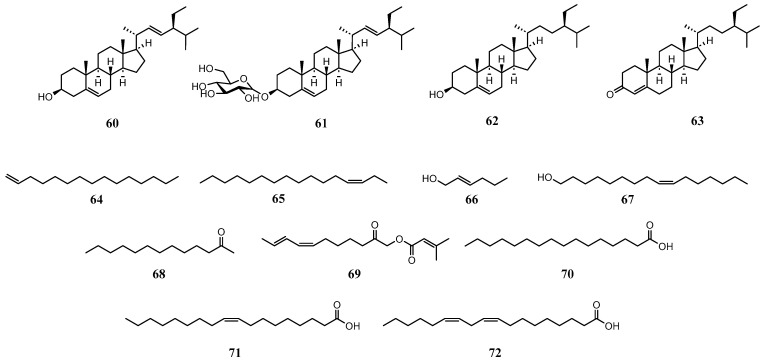
Structures of miscellaneous lipophilic compounds in *A. oleracea*.

In addition to the widely studied alkylamides, monoterpenes and sesquiterpenes in essential oils, and phenolic compounds investigated for their antioxidant properties, several other lipophilic compounds have been identified in *A. oleracea* (Figure 6 and Table 3). These primarily include substances with a steroid backbone (**60**–**63**) and various hydrocarbons along with their oxidized derivatives (**64**–**72**). Notably, stigmasterol (**60**), stigmasteryl-3-O-β-d-glucopyranoside (**61**), and β-sitostenone (**63**) were isolated through antioxidant and antimicrobial activity-guided fractionation [39]. Furthermore, numerous hydrocarbons and their derivatives are frequently identified in essential oils via GC-MS analysis [11,35,37,57]. Among these, (Z)-9-hexadecen-1-ol (**67**) exhibited a high relative abundance of 80.4% in the total ion chromatogram (TIC) [11].

Finally, Phrutivorapongkul et al. reported on the composition of fixed oils in *A. oleracea*. In the field of food and nutrition, it is well-established that the degree of unsaturation and the specific types of fatty acids within vegetable oils play a crucial role in determining their food industrial applications and ethno-pharmacological benefits [81,82]. According to the report from Phrutivorapongkul et al., the fixed oil fraction of *A. oleracea* is predominantly composed of alpha-linolenic acid (**72**) (56.37%), an essential omega-3 (ω-3) fatty acid. This was followed by palmitic acid (**70**) (25.85%), a saturated fatty acid, and oleic acid (**71**) (8.72%), a monounsaturated fatty acid [38]. These findings suggest that *A. oleracea* possesses superior nutritional value due to its high content of unsaturated fatty acids, particularly polyunsaturated fatty acids (PUFAs) like alpha-linolenic acid.

## 6. Biological Activities

*A. oleracea*, which is utilized globally both as a botanical and in various nutraceutical forms, has been the subject of extensive modern pharmacological investigations that align with its diverse traditional applications. Extensive research has identified a broad spectrum of pharmacological activities for *A. oleracea*, including anti-inflammatory, antioxidant, analgesic, and antimicrobial effects, as well as vasorelaxant, antiarrhythmic, and wound healing properties. These findings strongly validate the plant’s traditional applications, which have guided modern scientific inquiry into its multifaceted therapeutic value. A comprehensive summary of these activities is provided in Table 6.

### 6.1. Anti-Inflammatory Activities

Unresolved inflammation acts as a primary catalyst for various pathologies. The chronicity of the inflammatory process poses a fundamental danger, as it triggers a self-perpetuating cycle where inflammatory tissue damage leads to necrosis, which in turn further exacerbates the inflammatory response [97]. Traditionally, *A. oleracea* has been utilized to treat respiratory conditions such as throat complaints and tuberculosis [34]. In a study by Kim et al., the protective effects and underlying mechanisms of a methanol extract from the whole *S. acmella* plant were evaluated using an LPS-induced lung injury mouse model. Administration of the extract at doses of 1 and 10 mg/kg resulted in a dose-dependent inhibition of inflammation, as confirmed by histological analysis. Furthermore, the extract suppressed neutrophilic lung inflammation by reducing the mRNA expression of proinflammatory cytokines (IL-1β, IL-6, and TNF-α) and lowering the activity of myeloperoxidase (MPO), a key inflammatory marker in neutrophils. To further elucidate the molecular mechanisms, in vitro assays using RAW 264.7 cells demonstrated that the extract dose-dependently inhibited the nuclear localization of NF-κB and decreased the expression of NF-κB-dependent cytokine genes (IL-6 and IL-1β). Simultaneously, it enhanced the antioxidant defense by inhibiting the ubiquitination of Nrf2, thereby increasing nuclear Nrf2 levels and the expression of Nrf2-dependent genes such as NQO1. These findings collectively suggest that *A. oleracea* mitigates lung inflammation through the dual regulation of NF-κB inhibition and Nrf2 activation [83].

Another study reported the anti-inflammatory activity of an aqueous extract from the aerial parts using a carrageenan-induced rat paw edema model. While the positive control, aspirin (100 mg/kg), showed an inhibition rate of 63.1%, the extract demonstrated paw edema inhibition of 52.6%, 54.4%, and 56.1% at doses of 100, 200, and 400 mg/kg, respectively. These results suggest that hydrophilic compounds, such as polysaccharides and phenolic substances, may have significant anti-inflammatory potential [26]. In a study comparing the anti-inflammatory activities of different fractions, an 85% EtOH extract from *A. oleracea* flowers was subjected to sequential partitioning to obtain n-hexane, CHCl_3_, ethyl acetate, and n-butanol fractions. Their ability to inhibit LPS-induced NO production in RAW 264.7 cells was evaluated. The results indicated that at a concentration of 80 μg/mL, the CHCl_3_ fraction exhibited the highest potency with 85% inhibition of NO production, followed by the n-hexane fraction (72%), ethyl acetate fraction, and n-butanol fraction in descending order. Regarding spilanthol (**6**) (45, 90, and 180 μM), the most widely recognized active compound, it dose-dependently inhibited the mRNA expression and protein production of LPS-induced iNOS (inducible nitric oxide synthase) and COX-2 (cyclooxygenase-2) in RAW 264.7 cells. Furthermore, it significantly suppressed the secretion of proinflammatory cytokines, such as IL-1β and IL-6. Mechanistically, spilanthol (**6**) was found to inhibit the LPS-induced phosphorylation of cytoplasmic IκB and the DNA binding activity of NF-κB in a dose-dependent manner. These findings indicate that, consistent with the previously reported results about inflammatory activity at extract or fraction level, the anti-inflammatory efficacy of spilanthol (**6**) is mediated through the inactivation of the NF-κB signaling pathway [84].

Huang et al. reported the inhibitory effects of spilanthol (**6**) on allergic inflammation using a DNCB-induced atopic dermatitis mouse model. Treatment with spilanthol (**6**) at doses of 5 and 10 mg/kg significantly suppressed the elevation of IgE and IgG2a, which are key markers of allergic reactions. Furthermore, it was found to modulate the Th1/Th2 imbalance by increasing IgG1 levels. These therapeutic effects were attributed to the inhibition of the MAPK signaling pathway, specifically by suppressing the activation of ERK, JNK, and p38 proteins. Additionally, spilanthol (**6**) administration reduced epidermal thickness, collagen accumulation, and the infiltration of mast cells and eosinophils. Collectively, the study demonstrated that spilanthol (**6**) ameliorates allergic inflammation in DNCB-induced atopic lesions by improving mast cell infiltration, modulating Th1/Th2 cytokine levels, and inhibiting MAPK signaling. Additionally, spilanthol (**6**) administration reduced epidermal thickness, collagen accumulation, and the infiltration of mast cells and eosinophils. Collectively, the study demonstrated that spilanthol (**6**) ameliorates allergic inflammation in DNCB-induced atopic lesions by improving mast cell infiltration, modulating Th1/Th2 cytokine levels, and inhibiting MAPK signaling [85].

### 6.2. Antioxidant Activities

Due to its rich content of phenolic compounds, numerous studies have reported the antioxidant potential of *A. oleracea*. Wangsawatkul et al. evaluated the antioxidant capacities of the aerial parts extracted with various solvents (n-hexane, CHCl_3_, ethyl acetate, and MeOH) using DPPH radical scavenging and SOD (superoxide dismutase) activity assays. At a concentration of 200 μg/mL, the ethyl acetate (EA) and methanol (MeOH) extracts exhibited similarly potent DPPH radical scavenging activities at 47.90% and 47.76%, respectively, followed by the CHCl_3_ and n-hexane extracts. In contrast, when SOD activity was assessed via NBT (nitroblue tetrazolium) inhibition, the CHCl_3_ extract demonstrated the highest activity (57.92%). This was followed by the MeOH (47.02%) and EA (33.05%) extracts, while the n-hexane extract showed negligible activity (0.41%) [86]. Prachayasittikul et al. conducted experiments under nearly identical conditions and reported a consistent order of efficacy among the extracts. Notably, they reported the isolation and identification of four phenolic compounds (**44**, **46**, **47**, **59**), one terpenoid (**41**), and three steroids (**60**, **61**, **63**) from the active fractions. However, the biological activities of these individual isolated compounds were not further evaluated [39].

In a study investigating the activity of different plant parts and solvent extracts, the DPPH radical scavenging activity was found to be highest in the flowers, followed by the stems and leaves. Regarding the solvents used, the methanol extract exhibited the greatest potency, followed by the acetone and water extracts, respectively [87]. Meanwhile, Fajardo et al. comprehensively evaluated the efficacy of the methanol extract from *A. oleracea* leaves. At a concentration of 300 μg/mL, the extract reduced IFN-γ and LPS-induced ROS production in macrophages by approximately 69.03%. In the NO scavenging assay, it exhibited an IC_50_ value of 127.6 μg/mL for the inhibition of NO production. Furthermore, the extract showed a 63.69% inhibition rate regarding the production of MDA, a byproduct of lipid peroxidation. In addition to these findings, the study provided insights into the correlation between potent antioxidant capacity and phenolic content through total phenolic content (TPC) determination and compositional analysis [88].

At the single compound level, the efficacy of known active substances, vanillic acid (**44**) and trans-ferulic acid (**46**), has been reported in SH-SY5Y neuronal cell model where neurotoxicity was induced by H_2_O_2_. Both **44** and **46** were found to inhibit apoptosis and reduce ROS levels. Furthermore, pretreatment with these phenolic compounds effectively counteracted the H_2_O_2_-driven decline of SIRT1 and FoxO3a expressions in SH-SY5Y cells. This molecular upregulation was accompanied by an increase in key antioxidant enzymes, such as SOD2 and catalase, as well as the anti-apoptotic protein Bcl-2, thereby reinforcing the cellular defense against oxidative damage [89].

### 6.3. Analgesic Activity

A study investigating the analgesic properties of the aqueous extract of *A. oleracea* reported significant results in multiple pain models. In acetic acid-induced writhing tests using albino mice, administration of the extract at doses of 100, 200, and 400 mg/kg resulted in protection rates of 46.9%, 51.0%, and 65.6%, respectively. Although these values were slightly lower than the 79.7% protection rate of the positive control, aspirin (100 mg/kg), they nonetheless demonstrate substantial analgesic efficacy. Furthermore, in the tail flick test, the extract was found to significantly increase the pain threshold throughout the entire observation period (30 min, 1, 2, and 4 h post-administration) [26]. Dallazen et al. provided critical insights into the mechanistic aspects of the antinociceptive properties of *A. oleracea*. The researchers evaluated the effects of an alkylamide-rich n-hexane fraction from the flower ethanol extract using the formalin test at two distinct concentrations: an antinociceptive dose (5 μg/mL) and a pronociceptive dose (1.5 mg/mL). At the low dose, the extract significantly inhibited glutamate-induced pain in both the neurogenic and inflammatory phases. This effect was independent of the endogenous opioidergic system but was closely associated with TRPV1 modulation. Conversely, the pain-inducing behavior observed at the high dose was attenuated by the activation of the opioidergic system, treatment with TRPA1 antagonists, and the desensitization of TRP nociceptive fibers. Collectively, these findings demonstrate that alkylamides can exert contrasting biphasic effects depending on the dosage and provide specific information regarding the receptors involved [90].

A study reporting the antinociceptive activity of MeOH extracts (100 mg/kg) from different plant parts found that in the neurogenic phase of the formalin test, the aerial part extract exhibited stronger activity than the flower extract. Conversely, in the inflammatory phase, the flower extract showed greater potency than the aerial part extract. Both extracts demonstrated efficacy comparable to that of indomethacin. Furthermore, results from the open field and catalepsy tests showed no significant differences compared to the control group. This indicates that the extracts did not induce hypolocomotion or catalepsy, which are potential side effects typically associated with cannabinoid receptor agonists [91].

### 6.4. Anesthetic Activity

Chakraborty et al. validated the anesthetic efficacy of the aqueous extract of *A. oleracea* through in vivo testing. An intracutaneous wheal test conducted in guinea pigs at two concentrations, 10% and 20%, demonstrated dose-dependent anesthetic effects of 70.36% and 87.02%, respectively, compared to only 4.16% in the negative control group. Furthermore, in a plexus anesthesia test using frogs, the 20% concentration showed a mean anesthetic onset time of 5.33 min. Considering the negative control’s onset time of 24.15 min, these results indicate substantial anesthetic potency [92]. Next, the potential of *A. oleracea* as a fish anesthetic was demonstrated in a study where a spilanthol-rich fraction, obtained via supercritical fluid extraction (SFE), was administered to juvenile tambaqui (*Colossoma macropomum*). The results showed that deep anesthesia was effectively achieved at all tested levels, with 20 mg/L yielding the most efficient induction (<3 min) and recovery (<5 min) times. While a minimal dose of 2 mg/L provided effective sedation, the overall stress response remained minimal; all physiological indicators fully recovered within 48 h, highlighting the potential of jambu extract as a safe and potent anesthetic agent [93].

### 6.5. Antimicrobial Activity

Mbeunkui et al. reported the antiplasmodial activity of *A. oleracea* and the potential synergistic effects among its constituents. An alkylamide-rich fraction, derived from the methanol extract of the flowers, was tested against the chloroquine-sensitive (D10) strain of *Plasmodium falciparum*, followed by bioactivity-guided fractionation and isolation. The IC_50_ values for the active fractions 2 to 5 were 14.91, 22.04, 26.17, and 12.21 μg/mL, respectively. Subsequently, the major compounds (**1**, **6**, **9**, and **17**) were isolated from these four fractions, yielding IC_50_ values of 54.03, 26.43, 29.34, and 33.73 μg/mL, respectively. A comparison between the fractions and their respective major compounds suggests a potential synergistic effect. Specifically, fraction 2, which contains **1** as its primary constituent alongside a balanced proportion of other alkylamides, including spilanthol (**6**), demonstrated significantly higher activity than fraction 3, which consists of over 95% spilanthol (**6**). Notably, although **1** itself was approximately twofold less active than **6**, its corresponding fraction (fraction 2) outperformed fraction 3, reinforcing the hypothesis that the coexistence of multiple alkylamides enhances the overall antiplasmodial efficacy [41].

On the other hand, several studies have reported the antimicrobial activity of *A. oleracea* against a diverse range of microbial strains. Specifically, the methanol extract from the leaves exhibited antibacterial activity against *E. coli*, *S. epidermidis*, MRSA, and *P. aeruginosa*, as well as antifungal activity against *C. albicans*, with MIC (Minimum Inhibitory Concentration) values ranging from 125 to 1000 μg/mL. Notably, when assessing adhesion inhibition against *S. aureus*, *P. aeruginosa*, and their mixed biofilms, the extract showed inhibition rates of 44.71%, 95.5%, and 51.83%, respectively. Furthermore, it demonstrated significant growth inhibition of 77.17% for *S. aureus* and 62.36% for *P. aeruginosa* [88].

Prachayasittikul et al. evaluated the antimicrobial activities of various solvent extracts (n-hexane, CHCl_3_, ethyl acetate, and methanol) from the aerial part of *A. oleracea* against an extensive range of microbial strains, including *C. diphtheriae*, *S. cerevisiae*, *S. pyogenes*, *B. subtilis*, *M. luteus*, *S. epidermidis*, and *P. shigelloides*. Overall, fractions derived from the CHCl_3_ and methanol extracts exhibited potent growth inhibition against most of the tested strains. For instance, these fractions yielded MIC values ranging from 64 to 256 μg/mL against *C. diphtheriae* and 128 to 256 μg/mL against *B. subtilis* [39].

### 6.6. Vasorelaxant Activity

Wongsawatkul et al. reported the vasorelaxant effects of various solvent extracts (n-hexane, CHCl_3_, ethyl acetate, and methanol) from *A. oleracea* using an in vivo model. After inducing contraction in the rat thoracic aorta with phenylephrine, the efficacy and underlying mechanisms of the extracts were evaluated through solo administration or co-treatment with a NOS (nitric oxide synthase) inhibitor or a COX (cyclooxygenase) inhibitor. The results showed that the maximal relaxation (R_max_) for the n-hexane, CHCl_3_, ethyl acetate, and methanol extracts were 65.67%, 96.64%, 81.64%, and 65.09%, respectively. Furthermore, the ED_50_ values were recorded at 0.361, 0.428, 0.076, and 0.955 ng/mL, respectively. These findings indicate that the ethyl acetate extract induced the most rapid vasorelaxation, while the CHCl_3_ extract exhibited the highest overall vasorelaxant potency [86].

Meanwhile, a study reporting the vasorelaxant effect of spilanthol (**6**) and its plausible mechanisms revealed that this effect was partly dependent on the presence of the endothelium. Furthermore, the vasorelaxation was significantly inhibited in the presence of inhibitors of nitric oxide (NO), hydrogen sulfide (H_2_S), and carbon monoxide (CO) synthesis. These findings suggest that spilanthol-induced vasodilation is mediated by complex mechanisms involving both gasotransmitters and prostacyclin signaling pathways [94].

### 6.7. Others

In addition to the aforementioned activities, other pharmacological properties such as wound healing, antipyretic, antiarrhythmic, and gastroprotective effects have been reported. Regarding wound healing, a scratch wound healing assay using L929 fibroblasts demonstrated that the methanol extract from *A. oleracea* leaves achieved 97.86% cell migration compared to the control group. This result provides a scientific basis for its traditional use in wound recovery [88].

The antipyretic activity of the aqueous extract from the aerial parts was evaluated in a yeast-induced pyrexia rat model. At doses of 100, 200, and 400 mg/kg, the extract significantly reduced body temperature from 1 to 3 h post-administration, performing comparably to the positive control, aspirin (300 mg/kg). However, unlike aspirin, the antipyretic effect did not persist at the 4 h mark. The authors attributed this transient effect to phenolic compounds, such as flavonoids, present in the fractions [92].

Furthermore, the antiarrhythmic activity of a spilanthol-rich fraction obtained via supercritical fluid extraction was investigated. Its electrophysiological effects and impact on epinephrine-induced arrhythmia were evaluated. At doses of 10, 15, and 20 mg/kg, the fraction maintained sinus rhythm and preserved cardiac intervals while significantly reducing the heart rate and R-R interval. These results were found to be comparable to those of lidocaine [95].

The gastroprotective effect of polysaccharides derived from *A. oleracea* has been reported, which is a relatively unique finding regarding both the material and its efficacy. In an ethanol-induced gastric ulcer rat model, the administration of rhamnogalacturonans (polysaccharides) dose-dependently reduced gastric lesions, with an ED_50_ of 1.5 mg/kg. The underlying gastroprotective mechanisms are hypothesized to involve: (1) the formation of a protective physical barrier by binding to the mucosal surface; (2) the suppression of aggressive factors, specifically acid and pepsin secretions; and (3) the enhancement of mucosal defense through stimulated mucus synthesis and effective radical scavenging activity [96].

da Rocha et al. demonstrated that oral treatment of hydroalcoholic extract of flower of *A. oleracea* affected the estrous cycle without altering folliculogenesis and fertility in an animal model, a result which supports the ethnopharmacological uses of the plant as a prominent aphrodisiac in India and Brazil [16].

## 7. Discussions

As previously mentioned, there have been reports of taxonomic misapplication regarding the identification of *A. oleracea* across different countries. Furthermore, several species within the genus *Acmella*, which contain spilanthol (**6**) to some extent, exhibit similar analgesic and anesthetic properties; thus, these species are often utilized for the same purposes, mainly for toothache pain relief, in some countries, which makes categorizing ethnobotanical uses strictly by country challenging [2,98].

Also, applications often vary significantly by region and ethnic group even within a single country. For instance, the ‘Thai toothache plant’, which is used for toothaches but often documented as *A. oleracea* in the literature, has been reported to treat as many as 14 different symptoms depending on the ethnicity [3]. However, when limited to *A. oleracea*, the most frequent application of the plant is for toothache relief. This pattern is consistently observed across numerous countries and diverse cultural backgrounds [2,15]. The next most frequent uses include sexual enhancement (aphrodisiac), treatment of dry mouth (xerostomia), and various gastrointestinal disorders such as colic and indigestion [2,16,99]. This primary usage is believed to be attributed to the anesthetic and analgesic activities of spilanthol (**6**), the most abundant bioactive compound in *A. oleracea*. The diversity of other applications likely reflects cultural differences in disease prevalence and the varying availability of alternative medicinal plants in different regions.

Meanwhile, not only the types and content of secondary metabolites, but also their bioavailability is a crucial factor for good efficacy. In terms of research on the bioavailability of the extract of *A. oleracea* or spilanthol (**6**), most studies have focused on transdermal or transmucosal behavior using a Franz diffusion cell system rather than systemic bioavailability through oral administration using an in vivo model. According to Veryser et al., who conducted comprehensive studies about mucosal and blood–brain barrier transport kinetics of spilanthol (**6**), it exhibited favorable intestinal permeability, as evidenced by its bidirectional (apical to basolateral and vice versa) transport across Caco-2 monolayers (P_app_: 5.2–10.2 × 10^−5^ cm/h), and this result was confirmed by in vivo oral absorption data in rats, where an elimination rate constant k_e_ was 0.6 h^−1^. Notably, the compound demonstrated a remarkable capacity to penetrate the blood–brain barrier. Following systemic absorption, it showed a rapid brain uptake with a unidirectional influx rate constant K_1_ of 796 μL/(g·min) [100]. According to a study investigating the diuretic mechanism of spilanthol (**6**) in a mouse model, oral administration of spilanthol (**6**) (800 mg/kg) induced a significant increase in both urine output and salt urinary excretion associated with a markedly reduced urine osmolality compared with control mice. These findings indicate that spilanthol (**6**) is absorbed from the gastrointestinal tract and reaches the kidneys in sufficient concentrations, thereby indirectly supporting that spilanthol (**6**) has systemic bioavailability to some extent [101]. In addition, Jayashan et al. has reported that spilanthol (**6**) exhibited favorable characteristics for oral delivery, blood–brain barrier permeability, and minimal toxicity in silico pharmacokinetic and toxicity screening [102].

In terms of clinical studies, Pradhan et al. conducted a longitudinal study among 240 male participants consuming SA3X capsules (containing 500 mg of *S. acmella* extract, standardized to 3.5% spilanthol (**6**), delivering 17.5 mg spilanthol (**6**)) to determine the effect of *S. acmella* on muscle mass and sexual frequency over two months. Evaluations were performed at three time points at recruitment, at the end of three weeks, at the end of two months. Interestingly, there were statistically significant increases in mid upper-arm circumference (*p* = 0.050), frequency of sexual intercourse (*p* = 0.028), duration of penile erection (*p* = 0.032) after three weeks. Two months later, not only the three indicators mentioned above, but also chest circumference (*p* = 0.048) and thigh circumference (*p* = 0.036) were also statistically increased. These results provide scientific evidence of traditional uses of the plant as a potent aphrodisiac and for development of various therapeutic products [99].

In other clinical studies, anti-wrinkle emulsion serum loaded with *A. oleracea* extract in various formulations has been proved as safe, effective and non-invasive [103]. Also, efficacy and safety of a health supplement containing extracts of *A. oleracea* and *Boswellia serrata* as add-on therapy was evaluated among participants with chronic low back pain in an observational, real-world cohort study [104]. Another group reported that food-grade lecithin formulation of standardized ginger and *A. oleracea* extract was effect and free of side effects in patients with moderate knee osteoarthritis [105].

As a medicinal plant with a long history of safe use, the extract of *A. acmella* or spilanthol (**6**) has been developed into various formulations in neutraceutical or cosmetic industries. According to the European Food Safety Authority (EFSA), the recommended intake of spilanthol (**6**) as a flavoring substance is 24 mg/capita/day based on the Maximized Survey-derived Daily Intake (MSDI) approach [106]. Moreover, several products containing extracts of *A. oleracea* (syn. *S. acmella*), with spilanthol (**6**) as a major bioactive compound, are commercially available in various countries. For example, oral care products such as oral gels (Indolphar^®^ from I.D. Phar, Haaltert, Belgium) or toothpaste (Swissdent^®^ from SWISSDENT Cosmetics AG, Zurich, Switzerland) are marketed to alleviate oral pain and inflammation associated with dental conditions such as toothache [107,108].

Furthermore, several anti-aging products formulated as diluted extracts or gels (e.g., Gatuline^®^ from Gattefossé, Saint-Priest, France and Nativilis Jambu Spilanthol Gel from Nativilis Limited, London, UK) are available [109,110]. These products are in high demand, particularly among middle-aged women, as interest in beauty increases in both developing and developed countries and the efficacy of *A. oleracea* in safely reducing facial wrinkles has been scientifically proven, earning the reputation of ‘natural botox’ [103,111].

Additionally, tinctures of the plant (A. Vogel *Spilanthes* tincture containing 67% alcohol, from Biohorma, Elburg, The Netherlands) have been utilized for the topical treatment of fungal infections such as ringworm or athlete’s foot, or for oral treatment of candidiasis (thrush) with a recommended dose of 20 drops twice daily in a little water [4,112]. In Italy, Joint health supplement (Nervana^®^ from Sanitas Famaceutici, Tortona, Italy) containing standardized extracts of *A. oleracea* and *B. serrata* have been for sale [104].

## 8. Conclusions

Through this review, *A. oleracea* has been reaffirmed as a valuable natural resource that combines rich ethnopharmacological traditions with modern scientific evidence. N-alkylamides, led by the key constituent spilanthol (**6**), along with phenolic compounds, possess multifaceted therapeutic potential, including anti-inflammatory, analgesic, and anesthetic effects. This potential is translating into active patent filings and commercialization in industrial sectors such as oral care products and cosmeceuticals. Unlike previous reviews that addressed the entire genus broadly, this study distinguishes itself by systematically collecting the latest research findings specific to *A. oleracea* and organizing compounds selected for their high quantitative reliability. Such an integrative analysis will contribute to researchers identifying existing knowledge gaps and establishing more effective extraction processes and formulation strategies.

Future research should involve clinical trials based on the various pharmacological mechanisms presented in this review, alongside more in-depth standardized marker compound management for industrial mass production and long-term toxicological assessments. It is expected that this paper will serve as a significant milestone in the development of high-value natural medicines and functional materials utilizing *A. oleracea*.

## Figures and Tables

**Table 1:** Taxonomy of *A. oleracea*.

Taxa	Name
Kingdom	Plantae
Subkingdom	Tracheobiont
Phylum	Tracheophyta
Division	Magnoliophyta
Superdivision	Spermatophyte
Class	Magnoliopsida
Subclass	Asteridae
Order	Asterales
Family	Asteraceae
Subfamily	Mimosoideae
Genus	Acmella
Species	Oleracea

**Table 2:** Traditional application of *A. oleracea*.

Part	Traditional Uses (Country)	Ref.
Medicinal		
F	Toothache (Many countries in America, Asia and Africa such as Peru, Brazil, India, etc.), Bleeding, Stammering, Xerostomia (India), Sexual dysfunction (Brazil, India)	[14,15,16,17,20,32,33]
F & L	Tuberculosis (Brazil), Leucorrhoea (Bangladesh)	[21,22,24,26,34]
WP	Poisonous sting (Bangladesh), Rheumatism (India, Cameroon), Snakebite (Cameroon), Stomatitis (Indonesia), Urolithiasis, Digestive problems, Scurvy,	
R	Throat problem (India), Constipation	[18,24]
Culinary		
F	Used in curries (Taiwan), Spice (Japan), Additives in drinks, cocktails	[27,29,30,31]
L	Raw or vegetable (India, Brazil), Pungent flavoring for salads (U.S.), Served raw in ‘lalab’ with other vegetables and eaten with a sambal (chili sauce) (Indonesia)	[25,26,27,28]

F: Flower; L: Leave; WP: Whole plant.

**Table 3:** Summary of the isolated or identified secondary metabolites with medium to large amounts from *A. oleracea*.

Compound	Formula	Part	Extraction	Identification	Remark	Ref.
Alkylamide						
(2Z)-N-isobutyl-2-nonene-6,8-diynamide (**1**)	C_13_H_17_NO	F	MeOH ex.	HPLC, ESI-IT-TOF-MS, NMR	20 mg	[41]
(2E)-N-isobutyl-2-decamonoenamide (**2**)	C_14_H_27_NO	AP, R	80% EtOH ex., sonication	HPLC-DAD-ESI-MS	From in vitro seedlings	[42,43]
(2E,7Z)-N-isobutyl-2,7-decadienamide (**3**)	C_14_H_25_NO	AP	80% EtOH ex., sonication	HPLC-DAD-ESI-MS	From in vitro seedlings	[40]
(2E,6Z)-N-isobutyl-8,9-dihydroxy-2,6-decadienamide (**4**)	C_14_H_25_NO_3_	L	EtOH ex., CH_2_Cl_2_ fr.	NMR	15 mg of mixture with **3** out of 0.8 kg raw material	[44]
(2E,7E)-N-isobutyl-6,9-dihydroxy-2,7-decadienamide (**5**)	C_14_H_25_NO_3_	L	EtOH ex., CH_2_Cl_2_ fr.	NMR	15 mg out of 0.8 kg raw material	[44]
(2E,6Z,8E)-N-isobutyl-2,6,8-decatrienamide (spilanthol) (**6**)	C_14_H_23_NO	F	Hx ex.	GC-MS, NMR	135 mg out of 1.5 kg raw material	[45]
		F, L, S	SFE	GC-FID	65.4% (F), 19.7% (L), 47.3% (S) of dry weight	[46]
(2Z)-N-isobutyl-2-decene-6,8-diynamide (**7**)	C_14_H_19_NO	R	Petrol:Et_2_O (1:1)	HPLC, HR-MS, NMR	1 mg out of 50 g of raw material	[47]
(2E,7Z,9E)-N-isobutyl-2,7,9-undecatrienamide (**8**)	C_15_H_25_NO	F	Hx ex.	GC-MS, NMR	135 mg out of 1.5 kg	[45]
(2E)-N-isobutyl-2-undecene-8,10-diynamide (**9**)	C_15_H_21_NO	F	Hx ex.	NMR	5 mg out of 1.5 kg	[45]
(2E,4Z,8Z,10E)-N-isobutyl-2,4,8,10-dodecatetraenamide (**10**)	C_16_H_25_NO	A	Hx ex., soxhlet	GC-MS	67% of relative abundance	[11]
(2E,4Z)-N-isobutyl-2,4-undecadiene-8,10-diynamide (**11**)	C_15_H_19_NO	L	EtOH ex.	HPLC-ESI-MS/MS	15.8% of relative abundance	[48]
(2E,5Z)-N-isobutyl-2,5-undecadiene-8,10-diynamide (**12**)	C_15_H_19_NO	F	95% EtOH ex., EtOAc fr.	NMR, HR-ESI-MS	2.5 mg out of 4.5 kg of raw material	[49]
(2E,4E,8Z,10E)-N-isobutyl-2,4,8,10-dodecatetraenamide (**13**)	C_16_H_25_NO	AP	95% EtOH ex.	NMR, Q-TOF-MS	10 mg out of 1.5 kg of raw material	[38]
(2E,4E,8Z,10Z)-N-isobutyl-2,4,8,10-dodecatetraenamide (**14**)	C_16_H_25_NO	L	EtOH ex.	HPLC-ESI-MS/MS	38.0% of relative abundance	[48]
(7Z)-N-isobutyl-7-tridecene-10,12-diynamide (**15**)	C_17_H_25_NO	F	Hx ex.	HPLC, HR-MS	5 mg out of 200 g of Hx ex.	[50]
(2E,7Z)-N-isobutyl-2,7-tridecadiene-10,12-diynamide (**16**)	C_17_H_23_NO	F	Hx ex.	HPLC, HR-MS	5 mg out of 200 g of Hx ex.	[50]
						
(2E,6Z,8E)-N-(2-methylbutyl)-2,6,8-decatrienamide (homospilanthol) (**17**)	C_15_H_25_NO	F, L	EtOH ex.	HPLC-ESI-MS/MS	7.9% (F) and 12.7% (L) of relative abundance	[48]
(2E)-N-(2-methylbutyl)-2-undecene-8,10-diynamide (**18**)	C_16_H_23_NO	F	Hx ex.	HPLC, HR-MS	10 mg out of 200 g of Hx ex.	[50]
N-phenethyl-2,3-epoxy-6,8-nonadiynamide (**19**)	C_17_H_17_NO_2_	AP	PPO/water	UHPLC-DAD-ESI-MS/MS		[51]
N-phenethyl-2,3-dihydroxy-6,8-nonadiynamide (**20**)	C_17_H_19_NO_3_	L	EtOH ex., CH_2_Cl_2_ fr.	NMR	10 mg out of 0.8 kg raw material	[44]
(2Z)-N-phenethyl-2-nonene-6,8-diynamide (**21**)	C_17_H_17_NO	L	EtOH ex.	HPLC-ESI-MS/MS	15.5% of relative abundance	[48]
(2Z)-N-phenethyl-2-decene-6,8-diyamide (**22**)	C_18_H_19_NO	L	EtOH ex., Hx fr.	NMR		[44]
Monoterpenes						
Myrcene (**23**)	C_10_H_16_	F	HD	GC-MS	>10% of total volatile oil	[35]
(Z)-β-Ocimene (**24**)	C_10_H_16_	F	SD	GC-MS	14.0% of total volatile oil	[52]
Limonene (**25**)	C_10_H_16_	F	SD	GC-MS	23.6% of total volatile oil	[36,52]
β-Phellandrene (**26**)	C_10_H_16_	F	HD	GC-MS	>9% of total volatile oil	[35,36]
Thymol (**27**)	C_10_H_14_O	L, S	SD	GC-MS	18.3% of total volatile oil	[53]
						
β-pinene (**28**)	C_10_H_16_	F	HD	GC-MS	>10% of total volatile oil	[35,36]
Sesquiterpenes						
β-elemene (**29**)	C_15_H_24_	L	HD	GC-MS	4.53% of total volatile oil	[54]
Germacrene D (**30**)	C_15_H_24_	F, L	HD, SD	GC-MS	>10% of total volatile oil	[35,37]
α-Cadinol (**31**)	C_15_H_26_O	F, L	HD	GC-MS	2.2% of total detected area in GC-MS	[37]
γ-Cadinene (**32**)	C_15_H_24_	L, S	SD	GC-MS	13.3% of total volatile oil	[53]
Bicyclogermacrene (**33**)	C_15_H_24_	L	HD	GC-MS	2.15% of total volatile oil	[54]
Guaiol (**34**)	C_15_H_26_O	F	SD	GC-MS	>10% of total volatile oil	[35]
α-humulene (**35**)	C_15_H_24_	F, L, S	MAE (Hx)	GC-MS	10.4% of total volatile oil	[36]
(E)-Caryophyllene (**36**)	C_15_H_24_	F, AP	HD, SD	GC-MS	>20% of total volatile oil	[35,36,37]
Caryophyllene oxide (**37**)	C_15_H_24_O	F, L	SD	GC-MS	>20% of total volatile oil	[35,36]
Diterpene						
(E)-Phytol (**38**)	C_20_H_40_O	F, L	HD	GC-MS	2.2% of total detected area in GC-MS	[37]
Triterpene						
α-Amyrin (**39**)	C_30_H_50_O	WP	Light petrol, 90% EtOH	Crystallization, St: mp, [α]_D_, IR		[55]
β-Amyrin (**40**)	C_30_H_50_O	WP	Light petrol, 90% EtOH	Crystallization, St: mp, [α]_D_, IR		[55]
3-Acetylaleuritolic acid (**41**)	C_32_H_50_O_4_	AP	EtOAc et.	NMR, MS	23.7 mg out of 1 kg plants	[39]
Lupeol (**42**)	C_30_H_50_O	AP	Hx ex.	GC-MS	Detected in moderate amount	[11]
3-O-acetyl lupeol (**43**)	C_32_H_52_O_2_	AP	Hx ex.	GC-MS	Detected in moderate amount	[11]
Phenolic acid						
Vanillic acid (**44**)	C_8_H_8_O_4_	AP	EtOAc ex.	Recrystallization, NMR, MS	3.8 mg out of 1 kg plants	[39]
Caffeic acid (**45**)	C_9_H_8_O_4_	R	80% EtOH, sonication	HPLC-DAD-ESI-MS	From in vitro seedlings	[40]
Ferulic acid (**46**)	C_10_H_10_O_4_	AP	MeOH ex.	Recrystallization, NMR, MS	5.1 mg out of 1 kg plants	[39]
Isoferulic acid (**47**)	C_10_H_10_O_4_	AP	MeOH ex.	Recrystallization, NMR, MS	12.0 mg out of 1 kg plants	[39]
3-O-caffeoylquinic acid (**48**)	C_16_H_18_O_9_	R	80% EtOH, sonication	HPLC-DAD-ESI-MS	From in vitro seedlings	[40]
5-O-caffeoylquinic acid (**49**)	C_16_H_18_O_9_	R	80% EtOH, sonication	HPLC-DAD-ESI-MS	From in vitro seedlings	[40]
3,4-di-O-caffeoylquinic acid (**50**)						
3,5-di-O-caffeoylquinic acid (**51**)	C_25_H_24_O_12_	R	80% EtOH, sonication	HPLC-DAD-ESI-MS	From in vitro seedlings	[40]
4,5-di-O-caffeoylquinic acid (**52**)	C_25_H_24_O_12_	R	80% EtOH, sonication	HPLC-DAD-ESI-MS	From in vitro seedlings	[40]
Caffeoylmalic acid (**53**)	C_13_H_12_O_8_	AP	80% EtOH, sonication	HPLC-DAD-ESI-MS	From in vitro seedlings	[40]
Feruloylmalic acid (**54**)	C_14_H_14_O_8_	AP	80% EtOH, sonication	HPLC-DAD-ESI-MS	From in vitro seedlings	[43]
Petasiphenol (**55**)	C_18_H_16_O_7_	R	80% EtOH, sonication	HPLC-DAD-ESI-MS	From in vitro seedlings	[40]
Rosmarinic acid (**56**)	C_18_H_16_O_8_	R	80% EtOH ex., sonication	HPLC-ESI-MS, NMR	From in vitro seedlings	[43]
Flavonoid						
Miquelianin (**57**)	C_21_H_18_O_13_	L	80% EtOH ex., sonication	HPLC-DAD-ESI-MS	Peak was observed in UV chromatogram	[40,56]
Rutin (**58**)	C_27_H_30_O_16_	L	80% EtOH ex., sonication	HPLC-DAD-ESI-MS	Peak was observed in UV chromatogram	[40,56]
Coumarin						
Scopoletin (**59**)	C_10_H_8_O_4_	AP	MeOH ex.	Recrystallization, mp, NMR	4.0 mg out of 1 kg plants	[39]
Steroid						
Stigmasterol (**60**)	C_29_H_48_O	AP	Hx ex.	Recrystallization, mp	68.9 mg out of 1 kg plants	[39]
Stigmasteryl-3-O-β-d-glucopyranoside (**61**)	C_35_H5_8_O_6_	AP	CHCl_3_ ex.	Recrystallization, mp	15.4 mg out of 1 kg plants	[39]
β-sitosterol (**62**)	C_29_H_50_O	WP	Light petrol, 90% EtOH	St: mp, IR, co-TLC, NMR		[55]
β-sitostenone (**63**)	C_29_H_48_O	AP	EtOAc ex.	NMR, MS	4 mg out of 1 kg plants	[39]
Others						
1-pentadecene (**64**)	C_15_H_30_	F, L	HD	GC-MS	3.4% of total volatile oil	[35,37]
(Z)-3-pentadecene (**65**)	C_16_H_32_	WP	HD	GC-MS	4.5% of total volatile oil	[57]
(E)-2-hexenol (**66**)	C_6_H_12_O	WP	HD	GC-MS	11.0% of total volatile oil	[57]
(Z)-9-hexadecen-1-ol (**67**)	C_16_H_32_O	AP	Hx ex., soxhlet	GC-MS	80.4% of relative abundance	[11]
2-tridecanone (**68**)	C_13_H_26_O	WP	HD	GC-MS	13.1% of total volatile oil	[57]
(7Z,9E)-2-oxoundeca-7,9-dien-1-yl senecioate, Acmellonate (**69**)	C_16_H_24_O_3_	F, L	HD	GC-MS	4.7% of total detected area in GC-MS	[37]
Palmitic acid (C16:0) (**70**)	C_16_H_32_O_2_	AP	95% EtOH ex., maceration	GC	25.8% of fixed oil, raw material from Thailand	[38]
Oleic acid (C18:1 n-9) (**71**)	C_18_H_34_O_2_	AP	95% EtOH ex., maceration	GC	8.7% of fixed oil, raw material from Thailand	[38]
Linoleic acid (C18:2 n-6) (**72**)	C_18_H_32_O_2_	AP	95% EtOH ex., maceration	GC	56.4% of fixed oil, raw material from Thailand	[38]

WP: Whole plant; AP: Aerial part; F: Flower; L: Leave; S: Stem; ex.: Extract; fr.: Fraction; MAE: Microwave-assisted extraction; HD: Hydrodistillaion; SD: Steam distillation.

**Table 4:** Summary of the information on preparation of N-alkylamides from *A. oleracea*.

No.	Part (Origin)	Solvent	Extraction	Analysis	Contents	Remarks	Ref.
1	AP, R (in vitro seedling)	80% EtOH (1:20, *w*:*v*, 60 °C)	Sonication	HPLC-DAD-MS	Absolute amount of **6** and total NAAs (mg/g DE) AP: 2.20, 2,77 R: 0.22, 0.49	Samples were obtained from in vitro seedlings Samples were prepared after being defeated by Hx	[43]
2	F, L, S	MeOH (1:10, *w*:*v*, 100 °C)	ASE	HPLC-HRMS, NMR	Amount of **6** (mg/g, DW) and amphiphilics (%, DW) F: 16.50, 7.69 L: 0.34, 8.42 S: 0.24, 3.09	Comparison of **6** and amphiphilic compounds in different parts of *A. oleracea*	[64]
	F, L, S	CO_2_ (1:100, *w*:*v*, 323 K, 25 MPa)-CO_2_ with hydroethanolic enhancer	SFE-ESE	GC-FID	Yield of **6** (%, DB) F: 65.4 L: 19.7 S: 47.3	Development of optimized process for selective isolation of **6**	[46]
3	F, L (Brazil)	EtOH (1:5, *w*:*v*, RT)	Maceration	HPLC-ESI-MS/MS	Order of relative abundance (%) F: **6** (89.2), **17** (7.9), **3**, **9**, **16** L: **14** (38.0), **11** (15.8), **22** (15.5), **17** (12.7), **6** (7.6), **15** (3.8), **16** (3.2), **18**, **20**	Identification and comparison of major NAAs between ethanolic extraction of F and L	[48]
4	AP w/F (Italy)	Hx (1:10, *w*:*v*, 40 °C)	Sonication	HPLC-MS, NMR	Proportion of contents in Hx ex. (%): total NAAs (50.9), **6** (42.67), **17** (6.10), **3** (0.89), **9** (0.43), **16** (0.43)		[36]
5	AP w/F (Italy)	MeOH (1:10, *w*:*v*, RT)	Soxhlet	HPLC-DAD-IT-MS	Absolute amount (g/100 g, DB): **6** (1.9), **1** (<0.1), **3** (<0.1), **9** (<0.1), **17** (<0.1), **18** (<0.1)	Optimization of extraction solvent and method for **6** Contents comparison of **6** and major NAAs in different conditions Concentration of **6** and NAAs (Hx ex. > MeOH ex.) Extraction yield and absolute amount (Hx ex. < MeOH ex.)	[62]
6	AP w/F (Italy)	H_2_O	MAE, HD	GC-FID	Absolute amount of **6** in EO (g/100 g) MAE: 13.31 HD: 2.24	Development of method maximizing the content of **6** in EO	[36]
	F (Brazil)	95% EtOH (1:4)	Maceration	HPLC-ESI-MS	Absolute amount of **6** in ex. (mg/g) Crude ex.: 28.33 Activated charcoal treated ex.: 117.96	Increased the content of **6** and removed pigments by treatment of activated charcoal	[35]
7	Whole plant	65% EtOH (1:10, *w*:*v*)	-	HPLC-ESI- MS	Proportion among the total amount of NAA (%): **6** (88.8), **17** (9.0)	Extract (A. Vogel *Spilanthes* from Biohorma, Elburg, The Netherlands) Permeability for **6** in the 65% ethanolic extract is two times lower compared to 10 and 30% PG based extracts in transmucosal behavior	[61]
8	AP	Propane/H_2_O	-	UHPLC-DAD-ESI-MS/MS	**6**, **17** were the most stable NAA Photostability of NAA in various solutions: MeOH > EtOH > saline > H_2_O	Extract (from GATTEFOSSÉ, Saint-Priest, France) Evaluation of photostability of NAAs in various solutions	[51]

AP: Aerial part; F: Flower; L: Leave; S: Stem; R: Root; Hx: n-hexane; RT: Room temperature; ASE: Accelerated solvent extractor; SFE-ESE: Supercritical fluid extraction coupled with enhanced solvent extraction; MAE: Microwave-assisted extraction; HD: Hydrodistillaion; ex.: Extract; NAAs: N-alkylamides; DB: Dry biomass; DE: Dry extract; DW: Dry weight; EO: Essential oil; PG: Propylene glycol.

**Table 5:** Summary of the information on preparation of phenolic compounds from *A. oleracea*.

No.	Part (Origin)	Solvent	Extraction	Analysis	Contents	Remarks	Ref.
1	F (India)	Various factors (solvent, solvent ratio, temperature)	Soxhlet	Colorimetric method	Order of total phenolic content (mg GAE/g DM) Solvent: MeOH > Water > Ace > EtOH > Bu Solvent ratio: 50 > 30 > 40 > 20 > 10 Temperature: 50 > 60 > 40 > 30 Agitation speed: 500 > 400 > 300 > 200 > 100 pH: 5 > 4 > 6 > 7 > 8	Comprehensive kinetic study of various factors influencing the extraction of total phenolic contents (solvent, solvent ratio, temperature, agitation speed, pH)	[79]
2	F, L, S (Sri lanka)	MeOH (1:3)	Soxhlet	Colorimetric method	Total phenolic content (mg GAE/g, DM) : 5.34 (F), 7.59 (L), 1.65 (S)	Comparison of active metabolites in different parts of the plant	[9]
3	F, L, S (India)	80% EtOH (1:20, *w*:*v*, 60 °C)	Sonication	Colorimetric method	Total phenolic content (mg GAE/g, DW) FG: 1.98 (F), 3.19 (L), 1.37 (S) HG: 1.70 (F), 1.95 (L), 0.71 (S) Total flavonoid content (mg RE/g, DW) FG: 5.91 (F), 11.45 (L), 3.80 (S) HG: 7.98 (F), 9.10 (L), 3.38 (S)	Comprehensive comparison of active metabolites in different parts of the plant and different growing conditions	[56]
4	L (Sri lanka)	80% MeOH (1:50, *w*:*v*, 6 °C)	Maceration	Colorimetric method	Total phenolic content (mg GAE/g, DW) : 10.99 (FG), 11.45 (HG), 9.91 (CC) Total flavonoid content (mg RE/g, DW) : 11.33 (FG), 12.33 (HG), 7.38 (CC)	Comparison of active metabolites under different growing systems Total antioxidant capacity was proportional to total phenolic and flavonoid content	[10]
5	AP, R (in vitro seedling)	80% EtOH (1:20, *w*:*v*, 60 °C)	Sonication	HPLC-DAD-MS	Amount of total phenols (mg/g DE) AP: 8.68 R: 14.15 Order of amount of major phenols AP: **53**, **51**, **54**, **49**, **50**, **55**, **56** R: **51** (50% of total phenol), **50**, **49**, **56**	Samples were prepared after defatting by Hx	[43]

F: Flower; L: Leave; S: Stem, AP: Aerial part; R: Root; DM: Dry matter; DW: Dry weight; GAE: Gallic acid equivalent; RE: Rutin equivalent; FG: Field-grown; HG: Hydroponically grown; CC: Callus culture; Hx: n-hexane; Bu: n-BuOH; Ace: Acetone.

**Table 6:** Summary of biological activity of *A. oleracea*.

Activity	Cpd, ex., fr. (Part)	Assay/Cell Type/Model	Concentration	Result or Mechanism	Ref.
Anti-inflammatory	MeOH ex. (WP)	In vivo (C57BL/6 mice)- histology, RT-PCR, MPO assay	1, 10 mg/kg	(↓) Expression of IL-1, IL-6, TNF-α (↓) MPO activity (↓) Neutrophilic lung inflammation in histology	[83]
	MeOH ex. (WP)	In vitro (RAW264.7 cell lines)	10, 30 and 50 µg/mL	(↓) Nuclear localization of NF-κB (↓) Expression of NF-κB dependent cytokine genes (↓) Ubiquitination of Nrf2 (↑) Level of Nrf2 in the nucleus (↑) Expression of Nrf2-dependent genes	[83]
	Water ex. (AP)	In vivo (Albino Wistar rat)-paw edema test	100, 200 and 400 mg/kg	(↓) Paw edema at three doses (inhibition %: 52.6, 54.4, 56.1)	[26]
	Hx fr., CHCl_3_ fr., EA fr., Bu fr. from 85%EtOH ex. (F)	In vitro (RAW264.7 cell lines)	80 µg/mL	CHCl_3_ fr. and Hx fr. (↓) NO production significantly	[84]
	**6**	In vitro (RAW264.7 cell lines)-LPS induced inflammation	45, 90, 180 µM	(↓) LPS-induced iNOS and COX-2 mRNA and protein expression (↓) Proinflammatory cytokines (IL-1β, IL-6, TNF-α) (↓) LPS-induced p-IκB, NF-κB DNA binding activity	[84]
	**6**	In vivo (BALB/c mice)- DNCB induced AD-like skin lesions	5, 10 mg/kg	(↓) IgE, IgG2a, COX-2, iNOS expression via blocking MAPK pathway (↓) Epidermal thickness, collagen accumulation, mast cell, eosinophil infiltration	[85]
Antioxidant	Hx ex., CHCl_3_ ex., EA ex., MeOH ex. (AP)	In vitro-DPPH radical scavenging assay, SOD activity assay	200 µg/mL	Radical scavenging activity (%) : 4.90, 29.82, 47.90 and 47.76 (Hx ex., CHCl_3_ ex., EA ex., MeOH ex.) NBT inhibition (%) : 0.41, 57.92, 33.05 and 47.02 (Hx ex., CHCl_3_ ex., EA ex., MeOH ex.)	[86]
	Hx ex., CHCl_3_ ex., EA ex., MeOH ex. (AP)	In vitro-DPPH radical scavenging assay, SOD activity assay	300 µg/mL	Radical scavenging activity : EA ex. and MeOH ex. showed the most potent activity NBT inhibition : CHCl_3_ ex. exhibited the most potent SOD activity Activity guided isolation : Phenolic compounds (**44**, **46**, **47**, and **59**) and a terpenoid (**41**) and steroids (**60**, **61**, and **63**) from active fr.	[39]
	Hx fr., CHCl_3_ fr., EA fr., Bu fr. from 85% EtOH ex. (F)	In vitro-DPPH, ABTS radical scavenging assay	EC_50_	DPPH, ABTS radical scavenging assay : EA fr. showed strongest antioxidant activity (1.38, 3.32 µmol)	[84]
	Ace ex., MeOH ex., Water ex. (F, S, L)	In vitro-DPPH radical scavenging assay	EC_50_	Radical scavenging activity : MeOH ex. showed strongest activity, followed by acetone ex. and water ex. in all parts : ex. of F showed strongest activity, followed by ex. of S and L in MeOH ex.	[87]
	MeOH ex. (L)	In vitro (BALB/c peritoneal macrophages)- Evaluation of ROS level, DPPH, NO scavenging assay, MDA assay	Evaluation of ROS level : 18.75–300 µg/mL NO scavenging assay and DPPH radical scavenging assay : IC_50_ MDA assay : 7.5–30 µg/mL	Reduction in production of ROS (%) : 69.0 (300 µg/mL) IC_50_ (µg/mL) : 44.5 (DPPH) and 127.6 (NO) MDA assay : 63.69%	[88]
	**44** and **46**	In vitro (SH-SY5Y cells)- Cell viability, Carboxy-DCFDA assay	1, 5 μM	(↓) H_2_O_2_ induced toxicity, ROS level (↑) Expression of SIRT1 and FoxO 3a (↑) Levels of SOD2 and CAT, Bcl-2 proteins	[89]
Analgesic	Water ex. (AP)	In vivo (Swiss albino mice and Wistar albino rats)-Writhing test, tail flick test	100, 200 and 400 mg/kg	Protection from writhing (%) : 46.9, 51.0 and 65.6 Tail flick test : Increased the pain threshold significantly	[26]
	Hx fr. from EtOH ex. (F)	In vivo (male Swiss mice)-Chemical and sensorial test	5 µg/mL (antinociceptive) 1.5 mg/mL (pronociceptive)	: Exhibited dual effects depending on doses : Antinociceptive effect at low dose was not inhibited by opioid blocker, but by TRPV1 modulation : Pronociceptive effect at high dose was inhibited by opioid agonist, TRPA1 antagonist and TRP nociceptive fiber desensitization	[90]
	MeOH ex. (F and AP)	In vivo (both sexes Swiss mice)-Formalin test, open field test, and catalepsy test	100 mg/kg	Antinociceptive activity (neurologenic phase) : AP ex. > F ex. Antinociceptive activity (inflammatory phase) : F ex. > AP ex. Open field and catalepsy test (F ex.) : no difference compared to control	[91]
Anesthetic	Water ex. (AP)	In vivo (guinea pigs and frogs)-Intracutaneous wheal test and plexus anesthesia in frogs	10, 20%	Anesthesia effect (%) in intracutaneous wheal method : 70.36, 87.02 (10, 20% conc.) Mean onset of anesthesia (min) in Plexus Anesthesia Method : 5.33 (20% conc.):	[92]
	SFE ex. (F)	In vivo (fishes)-Determination of time of anesthetic induction and recovery	5–25 mg/L	Deep anesthesia at all conc. 20 mg/L is recommended for rapid induction (<3 min) and uneventful recovery (<5 min) Most blood parameters were returned within 48 h post-anesthesia	[93]
Antiplasmodial	MeOH ex. (F)	In vitro (chloroquine sensitive strain (D10) of *P. falciparum*)	IC_50_	IC_50_ (µg/mL) : 14.91, 22.04, 26.17 and 12.21 (active fraction 2–5) : 54.03, 26.43, 29.34 and 33.73 (Cpd **1**, **6**, **9**, **17**):	[41]
Antibacterial	MeOH ex. (L)	In vitro (*E. coli*, *S. epidermis*, *MRSA*, *P. aeruginosa*, *C. albicans*) - MIC, MBC, MFC - Bacterial killing assay - Evaluation of biofilms adhesion inhibition		MIC: 1000—125 µg/mL Adhesion of biofilm (%) : 44.71, 95.5 and 51.83 (*S. aureus*, *P. aeruginosa* and mixed) Growth inhibition (%) : 77.17, 62.36 (*S. aureus*, *P. aeruginosa*)	[88]
	Hx ex., CHCl_3_ ex., EA ex., MeOH ex. (AP)	In vitro (*C. diphtheriae*, *S. cerevisiae*, *S. pyogenes*, *B. cereus*, *B. subtilis*, *M. lutens*, *S. epidermis*, *P. shigelloides*) - MIC		Fr. from CHCl_3_ and MeOH ext. (↓) Growth of many tested organisms (e.g., *C. diphtheriae* MIC of 64–256 μg/mL and *B. subtilis* with MIC of 128–256 μg/mL)	[39]
Vasorelaxant	Hx ex., CHCl_3_ ex., EA ex., MeOH ex. (AP)	In vivo (male Sprague-Dawley rats) - Isometric tension measurement	ED_50_	Relaxation max (%) : 65.67, 96.64, 81.64 and 65.09 (Hx ex. CHCl_3_ ex., EA ex., MeOH ex.) ED50 (ng/mL) : 0.361, 0.428, 0.076 and 0.955 (Hx ex. CHCl_3_ ex., EA ex., MeOH ex.)	[86]
	6	In vivo (male Wistar rats) - Isometric tension measurement		Vasorelaxation was partly dependent on the presence of endothelium (↓) Vasorelaxation in the presence of inhibitors of NO, H_2_S, and CO synthesis (↑) Vasodilation by mechanisms that involve gasotransmitters and prostacyclin signaling pathways	[94]
Wound healing	MeOH ex. (L)	In vitro (L929 fibroblast) - Scratch wound healing assay with	18.75, 37.50 µg/mL	(↑) migration (97.86%)	[88]
Antipyretic	Water ex. (AP)	In vivo (albino rats)-yeast-induced pyrexia model	100, 200, 400 mg/kg	Antipyretic effect (↓) temperature of pyretic rats significantly from 1 h to 3 h	[91]
Antiarrhythmic	**6** rich fr. obtained by SFE	In vivo (male Wistar rats) - Assessment of cardiac electrophysiology - Epinephrine-induced arrhythmia	10, 15, 20 mg/kg	Maintained sinus rhythm and preserved cardiac intervals (↓) Reduction in heart rate and R-R interval significantly	[95]
Gastroprotective	Polysaccharide (L)	In vivo (female Wistar rats) - EtOH-induced gastric ulcers model	1, 3, 10, and 30 mg/kg ED_50_	(↓) EtOH-induced gastric lesion ED_50_ (mg/kg) : 1.5	[96]
Aphrodisiac	70% EtOH ex. (F)	In vivo (female Wistar rats) - biochemical, hematological analysis - macroscopic and histopathological anaysis	88.91, 444.57 mg/kg	(↑) Frequency of the proestrous and estrous phase w/o maternal toxicity	[16]

WP: Whole plant; AP: Aerial part; F: Flower; L: Leave; S: Stem; Cpd: Compound; ex.: Extract; fr.: Fraction; Hx: n-hexane; EA: Ethyl acetate; Bu: n-BuOH; Ace: Acetone; iNOS: inducible nitric oxide synthase; p-IκB: Phosphorylated inhibitor of kappa B; NF-κB: Nuclear factor kappa B; DNCB: 2,4-dinitrochlorobenzene; AD: Atopic dermatitis; IL-1β: Interleukin-1β; IL-6: Interleukin-6; COX-2: Cyclooxygenase-2; IgE: Immunoglobulin E; IgG2a: Immunoglobulin G2a; MAPK: Mitogen-activated protein kinase; DPPH: 2,2-diphenyl-1-picrylhydrazyl; SOD: superoxide dismutase; ROS: Reactive oxygen species; NO: Nitric oxide; Carboxy-DCFDA: Carboxy-2′,7′-dichlorofluorescein diacetate; conc.: Concentration; SFE: Supercritical fluid extraction; *P. falciparum*: *Plasmodium falciparum*; (↑): increased; (↓): decreased.

## Data Availability

No new data were created or analyzed in this study. Data sharing is not applicable to this article.
